# Leflunomide therapy for IgA vasculitis with nephritis in children

**DOI:** 10.1186/s12887-021-02866-y

**Published:** 2021-09-08

**Authors:** Ling Hou, Zhou Zhang, Yue Du

**Affiliations:** Department of Pediatrics, Shengjing Hospital of China Medical University, No. 36 Sanhao Street Heping District, 110004 Shenyang, China

**Keywords:** IgA vasculitis, Henoch-Schönlein purpura nephritis, Leflunomide, Children, Immunosuppressive agent, Corticosteroid

## Abstract

**Background:**

Henoch-Schönlein purpura (HSP), also called IgA vasculitis, is a systemic vasculitis characterized by deposits of immunoglobulin A in blood vessels. Renal impairment of these patients is the main determinant of prognosis. The optimal treatment of HSP nephritis (HSPN) in children remains controversial, but many clinicians administer an immunosuppressive agent with a corticosteroid. A previous study reported that leflunomide (LEF) with a corticosteroid was effective for adult patients with HSPN and nephrotic proteinuria. However, data on this treatment in pediatric patients is limited.

**Methods:**

We described our experience at a single center on the use of LEF in 5 pediatric patients who had IgA vasculitis with proteinuria that was nearly 50 mg/kg (nephrotic range) and remained high despite administration of intravenous steroid, and biopsy-proven nephritis. All patients had class II to IIIb lesions based on the International Study of Kidney Disease in Children (ISKDC).

**Results:**

We successfully treated all 5 children who had IgA vasculitis with nephritis using LEF with a corticosteroid. Four patients achieved a complete remission of proteinuria, and 1 patient had significantly reduced proteinuria. The children received LEF for 6 months to 12 months, and none of them had severe adverse events.

**Conclusions:**

To our knowledge, this is the first case series to report successful treatment of pediatric HSPN with LEF in combination with a corticosteroid.

## Background

Henoch-Schönlein purpura (HSP), also called IgA vasculitis, is a type of vasculitis in which small blood vessels accumulate deposits of immunoglobulin A_1_. HSP is more common in children [1] and is characterized by non-thrombocytopenic purpura, abdominal pain, joint swelling and pain, and hematuria or proteinuria [2]. Renal involvement is an important determinant of long-term prognosis [3–5]. About 30 to 90 % of HSP patients have mild and self-limiting proteinuria and hematuria, and these patients generally have a good prognoses [4–6]. However, an increase in the urine protein level or the development of nephrotic syndrome or rapidly progressive glomerulonephritis is indicative of poor prognosis [7, 8]. Aggressive measures are usually required to improve the long-term outcomes of these patients.

Currently, clinicians often treat these patients with a combination of corticosteroids and immunosuppressants [9], including cyclophosphamide (CTX) [10], mycophenolate mofetil (MMF) [11], cyclosporine A (CysA) [12], and azathioprine [13]. In our clinical experience, we found that many patients and families do not accept CTX therapy, mainly due to its gonadal toxicity. Although MMF is effective and safe, its high cost may impact treatment decisions. Leflunomide (LEF) is a relatively new oral immunosuppressant that is approved for treatment of rheumatoid arthritis and psoriatic arthritis, but has also been used for treatment of juvenile rheumatoid arthritis, lupus nephritis [14], IgA nephropathy [15], and other conditions. A previous study reported that LEF in combination with a corticosteroid led to good outcomes in adult patients with HSP who had nephrotic-range proteinuria [16]. In addition, LEF is less expensive than many other drugs used to treat HSPN and has a favorable safety profile. However, little is known about the effect of LEF in pediatric patients with HSPN. The present case series describes our experience in the use of LEF for 5 pediatric patients who had IgA vasculitis with nephritis.

## Methods

**Fig. 1:**
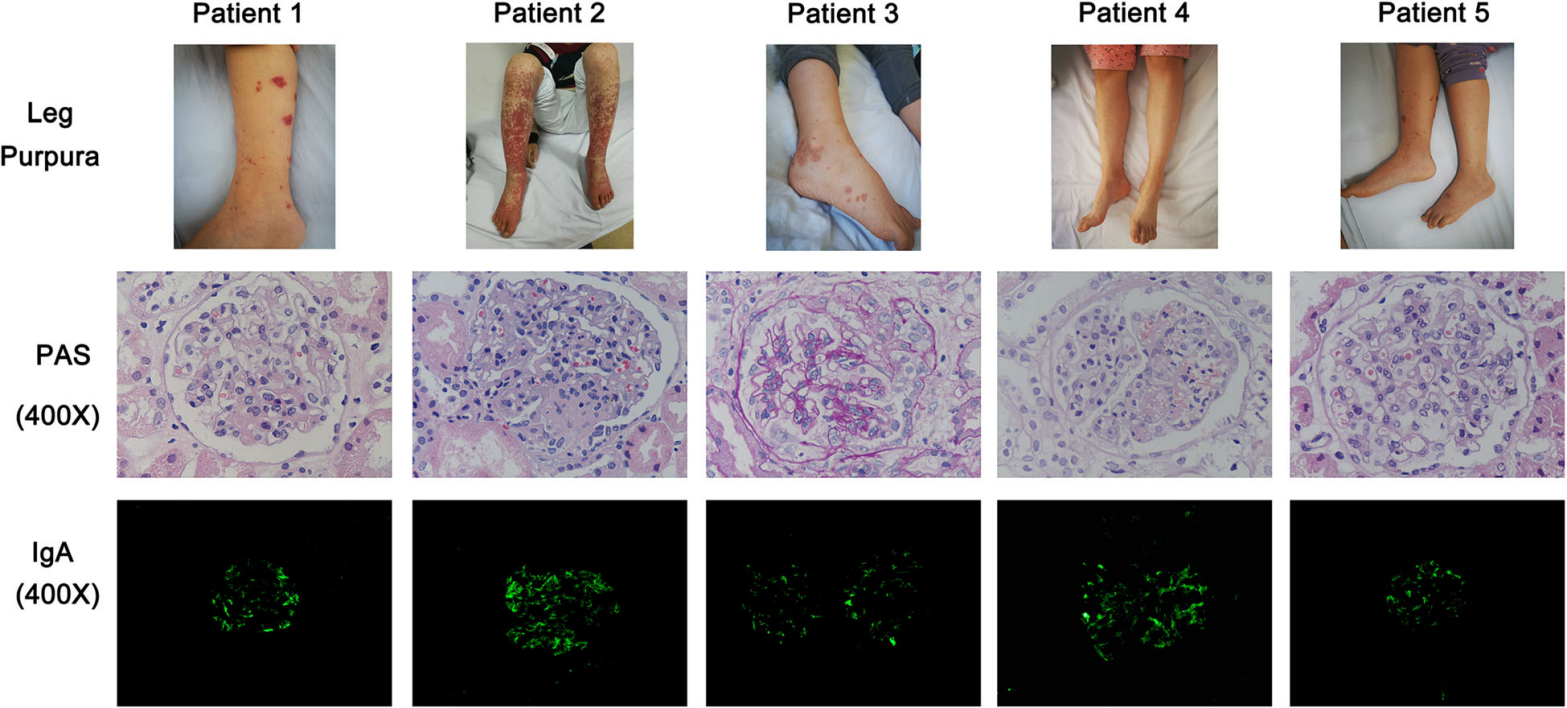
Leg purpura, and PAS staining and immunofluorescence analysis of IgA in renal tissues of 5 pediatric patients before LEF treatment

Five children (3 girls and 2 boys) who had diagnoses of IgA vasculitis with nephritis were first admitted to our department between February 2014 and April 2016 and received LEF treatment (Table 1; Fig. 1). The study was approved by the research ethics board of Shengjing Hospital of China Medical University (2016PS41J), and all methods were performed in accordance with the relevant guidelines and regulations. Informed consent was obtained from the parents or legal guardians. Diagnosis was based on EULAR/PRINTO/PRES criteria [17]. Urinary protein was defined as normal (≤ 5 mg/kg/day), pathological (5–50 mg/kg/day), or nephrotic (≥ 50 mg/kg/day). Children weighing 20 to 40 kg received 20 mg of LEF for 3 days followed by a maintenance dose of 10 mg per day; children weighing more than 40 kg received 40 mg of LEF for 3 days followed by a maintenance dose of 20 mg per day. These dosing schedules are based on the drug instruction for treatment of adult rheumatoid arthritis (50 mg for 3 days followed by a maintenance dose of 10–20 mg per day) and our experience of using leflunomide for juvenile idiopathic arthritis.

**Table 1:** Characteristics of patients at baseline and after LEF treatment

Patient #	Gender	Age	Body weight	Prior Mp therapy	Organ involvement	ISKDC grade	LEF treatment duration	Follow-up duration	Outcome
1	Female	12 years	55 kg	1.45 mg/kg/day, 12 days 2.18 mg/kg/day, 7 days	S, A, K	II	8 months	12 months	No skin rash UP: 8 mg/kg/day
2	Male	12 years	45 kg	1.78 mg/kg/day, 3 days 3.56 mg/kg/day, 8 days 11.11 mg/kg/day, 3 days	S, K	IIIb	7 months	8 months	No skin rash UP: undetectable
3	Female	11 years	35 kg	2.29 mg/kg/day, 5 days 4.57 mg/kg/day, 6 days	S, K, J	IIIb	12 months	18 months	No skin rash UP: undetectable
4	Female	11 years	30 kg	2.67 mg/kg/day, 6 days 5.33 mg/kg/day, 5 days 16.67 mg/kg/day, 3 days	S, K, J	IIIa	6 months	32 months	No skin rash UP: undetectable
5	Male	12 years	45 kg	1.78 mg/kg/day, 4 days 3.56 mg/kg/day, 8 days	S, K	II	6 months	7 months	No skin rash UP: undetectable

*A* abdomen, *ISKDC* International Study of Kidney Disease in Children, *K* kidney, *LEF* leflunomide, *Mp* methylprednisolone, *S* skin, *UP* urine protein, *J* joint

## Results

### Patient 1

In February 2014, a 12-year-old girl was admitted to our hospital for leg purpura, abdominal pain, microscopic hematuria, and proteinuria (25 mg/kg/day). The blood urea nitrogen (BUN) was 4.07 µmol/L, serum creatinine (SCr) was 51 mmol/L, serum albumin was 40.5 g/L, and estimated glomerular filtration rate (eGFR) was 116 mL/min/1.73 m^2^. A renal biopsy showed 21 glomeruli with diffuse moderate and focal mesangial cell proliferation and an increased mesangial matrix. Immunofluorescence (IF) analysis of the mesangial lesions indicated they were positive for IgA (predominant), IgM, and C3, leading to a diagnosis of HSPN ISKDC class II and M1 E0 S0 T0 C0 (revised Oxford classification ) (Fig. 1). We administered intravenous methylprednisolone (1.45 mg/kg/day for 12 days, followed by 2.18 mg/kg/day for 7 days), and this led to reduced abdominal pain and cutaneous vasculitis, but the proteinuria increased to 41 mg/kg/day. Because of the elevated proteinuria despite 19 days of intravenous steroid therapy, we administered LEF (40 mg/day for 3 days and followed by 20 mg/day for 8 months) and tapered the steroid to oral prednisone. We also administered an angiotension-converting enzyme inhibitor (captopril, 0.5 mg/kg, 2–3 times daily) for 3 months to reduce the proteinuria. The patient received follow-ups every 2 to 3 months. At the 12-month-follow-up, the patient had no recurrence of the skin rash or abdominal pain, normal clinical status and renal function, no hypertension, a urinary protein level of 8 mg/kg/day, 45 red blood cells (RBCs) per high power field (HPF), and no relevant adverse events (Table 1). This girl received oral prednisone for 12 months. Because her clinical status was stable, we scheduled follow-ups every 6 months.

### Patient 2

In July 2014, a 12-year-old boy was admitted to our hospital for microscopic hematuria and proteinuria (43 mg/kg/day). He had a history of leg purpura for 1 month. The BUN was 4.08 µmol/L, serum creatinine was 53.6 mmol/L, serum albumin was 40.7 g/L, and eGFR was 124 mL/min/1.73 m^2^. A renal biopsy isolated 51 glomeruli. Light microscopy indicated moderate-to-severe mesangial hypercellularity, expanded matrix, and three glomeruli with epithelial crescents. IF analysis indicated the mesangial lesions were positive for IgA (predominant), IgM, and C3, leading to a diagnosis of HSPN ISKDC class IIIb and M1 E0 S0 T0 C1 (revised Oxford classification ) (Fig. 1). We administered intravenous methylprednisolone (1.78 mg/kg/day for 3 days followed by 3.56 mg/kg/day for 8 days), but the proteinuria remained (52 mg/kg/day). We subsequently administered a pulse of intravenous methylprednisolone (11.11 mg/kg/day for 3 days) with subsequent oral prednisone, and this partially resolved the proteinuria (20 mg/kg/day), but also led to increased intraocular pressure (45 mmHg, measured with a noncontact tonometer for a weekly in hospitalized patients) with no symptoms. An intravenous steroid was used for 14 days, and we rapidly tapered the oral prednisone and administered LEF (40 mg/day for 3 days followed by 20 mg/day for 7 months). We also administered captopril (0.5 mg/kg, 2–3 times daily) for 3 months to reduce the proteinuria. The patient received follow-ups every 2 to 3 months. At the 8-month-follow-up, the patient had no recurrence of the skin rash, no detectable urinary protein, no relevant adverse events, normal clinical status and renal function, no hypertension (Table 1). He used oral prednisone for 6 months. Because his clinical status was stable, we scheduled follow-ups every 6 months.

### Patient 3

In April 2016, an 11-year-old girl was admitted to our hospital for leg purpura, bilateral swelling of the wrists, microscopic hematuria, and proteinuria (40 mg/kg/day). The BUN was 1.37 µmol/L, serum creatinine was 29.8 mmol/L, serum albumin was 39.2 g/L, and eGFR was 119 mL/min/1.73 m^2^. A renal biopsy provided 25 glomeruli. Light microscopy indicated diffuse moderate and focal severe mesangial cell proliferation and increased mesangial matrix, segmental endothelial cell proliferation, visceral and parietal epithelial swelling, two glomeruli with cellular crescents and two other glomeruli with small cellular crescents. There was also focal interstitial edema. IF analysis indicated the mesangial region was positive for IgA, and weakly positive for IgM and C3, leading to a diagnosis of HSPN ISKDC class IIIb and M1 E1 S0 T0 C1 (revised Oxford classification ) (Fig. 1). We administered intravenous methylprednisolone (2.29 mg/kg/day for 5 days followed by 4.57 mg/kg/day for 6 days), and this reduced the proteinuria to 20 mg/kg/day. We then progressively tapered the oral prednisone and administered LEF (20 mg/day for 3 days followed by 10 mg/day for 12 months) and captopril (0.5 mg/kg 2–3 times daily for 3 months) to reduce the proteinuria. The patient received follow-ups every 2 to 3 months. At the 18-month-follow-up, she had no recurrence of skin rash, no detectable urinary protein, no relevant adverse events, normal clinical status and renal function, no hypertension (Table 1). She used oral prednisone for 12 months. Because her clinical status was is stable, we scheduled follow-ups every 6 months.

### Patient 4

In November 2015, an 11-year-old girl was admitted to our hospital for leg purpura, bilateral swelling of the wrists and knees, microscopic hematuria, and proteinuria (25 mg/kg/day). The BUN was 4.26 µmol/L, and serum creatinine was 30.2 mmol/L, serum albumin was 37.9 g/L, and eGFR was 121 mL/min/1.73 m^2^. A renal biopsy was performed and 18 glomeruli were isolated. Light microscopy indicated focal moderate-to-severe mesangial hypercellularity with expanded matrix and segmental endothelial cell proliferation, visceral and parietal epithelial swelling, and two glomeruli with epithelial crescents and focal segmental necrosis. IF analysis indicated the presence of IgA (predominant), IgM, and C3 in the mesangial lesions, thus leading to a diagnosis of HSPN ISKDC class IIIa and M1 E1 S1 T0 C1 (revised Oxford classification ) (Fig. 1). We administered intravenous methylprednisolone (2.67 mg/kg/day for 6 days followed by 5.33 mg/kg/day for 5 days), and then a methylprednisolone pulse (16.67 mg/kg/day for 3 days) because of continuing proteinuria. However, the proteinuria increased to 42 mg/kg/day. We then administered LEF (20 mg/day for 3 days followed by 10 mg/day for 6 months) and tapered the steroid to oral prednisone. We also administered captopril (0.5 mg/kg, 2–3 times daily for 3 months) to reduce the proteinuria. The patient received follow-ups every 2 to 3 months. At the 32-month-follow-up, she patient had no recurrence of skin rash, no detectable urinary protein, no relevant adverse events, normal clinical status and renal function, and no hypertension (Table 1). She used oral prednisone for 9 months. Because of her stable clinical status, we scheduled follow-ups every 6 to 12 months.

### Patient 5

In September 2015, a 12-year-old boy was admitted to our hospital for leg purpura, microscopic hematuria, and proteinuria (34 mg/kg/day). The BUN was 3.82 µmol/L, serum creatinine was 39.4 mmol/L, serum albumin was 33.9 g/L, and eGFR was 120 mL/min/1.73 m^2^. A renal biopsy isolated 28 glomeruli and light microscopy indicated diffuse moderate to focal severe mesangial hypercellularity with expanded matrix and segmental endothelial cell proliferation, and visceral and parietal epithelial swelling. IF analysis indicated the presence of IgA (predominant) and C3 in the mesangial lesions, leading to a diagnosis of HSP ISKDC class II and M1 E1 S0 T0 C0 (revised Oxford classification ) (Fig. 1). We administered intravenous methylprednisolone (1.78 mg/kg/day for 4 days followed by 3.56 mg/kg/day for 8 days), and this reduced the proteinuria to 10 mg/kg/day. We then progressively tapered the patient to oral prednisone, and administered LEF (20 mg/day for 3 days followed by 10 mg/day for 6 months) and captopril (0.5 mg/kg, 2–3 times daily for 3 months) to reduce the proteinuria. Although this patient weighed 45 kg, his parents requested use of a lower dose of LEF, and this lower dose subsequently appeared to be effective. The patient received follow-ups every 2 to 3 months. At the 7-month-follow-up, he had no recurrence of the skin rash, undetectable urinary protein, no relevant adverse events, normal clinical status and renal function, and no hypertension (Table 1). He used oral prednisone for 6 months. Because of his stable clinical status, we scheduled follow-ups every 6 months.

## Discussion

We described 5 children who presented with IgA vasculitis with proteinuria that was nearly 50 mg/kg (nephrotic range) and remained high despite administration of intravenous steroid. All children had biopsy-proven nephritis, and we described their successful treatment with LEF combined with a corticosteroid. None of these patients received diuretics because of their relatively disease-free status (no nephrotic syndrome or oliguria). Two patients previously received intravenous steroid without resolution of proteinuria. Steroid therapy was quickly discontinued in 1 patient who experienced increased intraocular pressure. Four patients achieved complete renal remission, and the other patient had significantly reduced proteinuria. In all cases, remission persisted for the entire follow-up period of 8 to 32 months and there were no severe adverse events.

The optimal therapeutic strategy for IgA vasculitis in children remains controversial. Some patients only receive supportive therapy in an effort to control the acute symptoms. However, more intensive and specific treatment for IgA vasculitis with nephritis should be considered in pediatric patients with severe proteinuria and/or impaired renal function [18]. Although nephritis is the most serious long-term complication of IgA vasculitis, limited data are available regarding the best therapy. Corticosteroids are currently a first-line treatment for HSPN, and this approach leads to remission in most patients. However, some patients exhibit steroid dependence or steroid resistance, and require immunosuppressant therapy in combination with a corticosteroid.

The SHARE recommended treatments for children with moderate IgA vasculitis with nephritis include oral prednisolone and/or pulsed methylprednisolone as a first-line treatment, and AZA, MMF or intravenous CTX as a first- or second-line treatment [9]. Due to the lack of evidence-based data for treatment of IgA vasculitis with nephritis and the similarities of this condition with primary IgA nephropathy, the KDIGO guidelines proposed the same treatment for both diseases. Thus, they recommend oral or intravenous corticosteroids plus oral or intravenous CTX for initial therapy, but provided no recommendations for maintain therapy [10], and did not recommend MMF or AZA. Clinicians who strictly follow the KDIGO guidelines for the treatment of HSPN may face the risk of delayed initiation of effective treatment and an increased risk of chronic kidney disease over the long term.

The adverse effects of CTX, especially on the gonads, often reduce patient compliance. MMF (which selectively suppresses the proliferation of T and B cells) is another commonly used immunosuppressant. The adverse events from MMF therapy are less severe than those from CTX, but the high-cost of MMF restricts its use in low-medium income countries. CysA (which decreases lymphocyte function) is another commonly used immunosuppressant. CysA can be very effective in pediatric patients with severe HSPN; however, administration can be difficult because it has poor water solubility and close monitoring of the blood concentration is necessary. Cys A may also cause severe adverse events [19]. Azathioprine, a prodrug of 6-mercaptopurine, has antileukemic, anti-inflammatory, and immunosuppressive properties, and is also often used in the treatment of IgA vasculitis with nephritis [9, 13]. Previous studies reported administration of tacrolimus and rituximab for treatment of HSPN [20, 21], but there is limited evidence of their efficacy and further research is required for confirmation. LEF is an affordable medication that is associated with few adverse events. Research in adults with HSPN reported that LEF in combination with a corticosteroid may lead to a slower deterioration of GFR than corticosteroid monotherapy [16].

We administered LEF treatment to these patients because their parents refused CTX due to possible side effects and they could not afford MMF treatment. AZA was not availble in our hospital, and we had limited experience with AZA and CysA. LEF is commonly used for juvenile idiopathic arthritis and lupus nephritis in our center, and we found no obvious adverse effects, suggesting suitability for our patients. LEF is an immunosuppressive agent that disrupts T and B cell function *via* inhibition of dihydroorotate dehydrogenase (the rate-limiting enzyme of *de novo* pyrimidine nucleotide biosynthesis) and also inhibits several tyrosine kinase signaling molecules involved in immune function [22]. LEF is currently a common treatment for autoimmune diseases. There is evidence that LEF is effective in treating IgA nephropathy, and nephropathy caused by systemic vasculitis and systemic lupus erythematosus [14, 15]. In this study, we prescribed LEF in combination with a corticosteroid to successfully treat 5 children who had IgA vasculitis with nephritis. The duration of LEF therapy ranged from 6 to 12 months, and there were no severe adverse events.

This study is an observational case series. Data from randomized controlled trials (RCTs) supporting the use of LEF as a treatment for IgA vasculitis with nephritis are currently lacking. Thus, we need to study more patients using a well-designed RCT to compare LEF with conventional agents (i.e., a corticosteroid and a standard immunosuppressive agent) to confirm the effectiveness and safety of LEF therapy. Nonetheless, our examination of 5 children who had IgA vasculitis with nephritis indicated that LEF in combination with a corticosteroid achieved complete or nearly complete renal remission, and that none of the patients experienced severe adverse events.

## Data Availability

The datasets used and analysed during the current study are available from the corresponding author on reasonable request.

## Abbreviations

HSP: Henoch-Schönlein purpura
LEF: Leflunomide
CTX: Cyclophosphamide
MMF: Mycophenolate mofetil
Cys A: Cyclosporine A
SCr: Serum creatinine
